# Diferenças entre os Bloqueadores dos Receptores da Angiotensina (BRA) no Tratamento da Hipertensão Arterial

**DOI:** 10.36660/abc.20220281

**Published:** 2022-06-06

**Authors:** José Geraldo Mill

**Affiliations:** 1 Universidade Federal do Espírito Santo Departamento de Ciências Fisiológicas Vitória ES Brasil Departamento de Ciências Fisiológicas, Universidade Federal do Espírito Santo, Vitória, ES – Brasil; 2 Hospital Universitário Cassiano Antônio Moraes Vitória ES Brasil Hospital Universitário Cassiano Antônio Moraes, Vitória, ES – Brasil

**Keywords:** Hipertensão, Inibidores da Enzima Conversora da Angiotensina, Losartana, Diagnóstico Precoce

As doenças cardiovasculares (DCV) são a principal causa de morte e incapacidade no Brasil, e a hipertensão arterial (HA) é o principal fator de risco para morbimortalidade cardiovascular.^1^ O diagnóstico precoce e o tratamento correto são ações prioritárias para o enfrentamento do problema.^2^ A Pesquisa Nacional de Saúde realizada pelo Ministério da Saúde em 2013 (PNS-2013) determinou a prevalência de HA pela medida direta da pressão arterial (PA) e uso de anti-hipertensivos drogas em uma amostra representativa da população adulta brasileira. A prevalência de 32,3% (IC 95%: 31,7 - 33,0) indicou cerca de 50 milhões de hipertensos.^3^ Cerca de 70% dependem do Sistema Único de Saúde (SUS) tanto para diagnóstico quanto para assistência farmacêutica, aspecto essencial do plano de Doenças Crônicas Não Transmissíveis (DCNT).^2^

De acordo com as diretrizes atuais, o tratamento inicial da HA deve ser realizado com medidas gerais, incluindo atividade física aeróbica regular, redução da ingestão de sal, aumento do consumo de frutas e hortaliças e redução de peso na presença de obesidade ou sobrepeso.^4^ Essas medidas beneficiam a todos e não só aos hipertensos. Mesmo adotando essas estratégias, muitos pacientes ainda dependem do uso regular de medicamentos para o controle da PA elevada. Assim, o uso desses medicamentos apresenta grande importância, pois, dada a dimensão do problema, mesmo pequenas reduções pressóricas geram impacto positivo para milhões de indivíduos afetando as taxas de morbimortalidade por DCV.^5^ Assim, a busca por tratamentos eficazes para o controle da PA é de suma importância para a adoção de políticas públicas nessa área.

O SUS disponibiliza pelo menos um medicamento dentre as sete classes de anti-hipertensivos mais utilizados na rotina clínica, contribuindo para a alta cobertura medicamentosa em hipertensos no Brasil em comparação a outros países. Pesquisa nacional realizada em 2016 mostrou que 93,8% dos indivíduos que sabiam seu estado hipertensivo usavam pelo menos um anti-hipertensivo.^6^ Índices de tratamento elevados (>80%) também foram relatados na PNS-2013 e na coorte ELSA-Brasil, onde a maioria dos participantes é atendida por plano de saúde privado.^7,8^ Um achado importante na PNS foi mostrar que a frequência de uso independia da escolaridade e da renda, confirmando a universalidade do acesso, um dos objetivos da política nacional de enfrentamento DCNT no Brasil.^2^

Os bloqueadores dos receptores da angiotensina (BRA) são o anti-hipertensivo mais utilizado no Brasil.^7,8^ Após a introdução do losartana, composto protótipo do BRA no arsenal terapêutico da HA há mais de 30 anos, uma série de outros compostos com o mesmo mecanismo de ação foram disponíveis para uso. A eficácia desses compostos no controle da PA é o tema central do artigo de Barroso et al.,^9^ publicado nesta edição dos Arquivos Brasileiros de Cardiologia. Este robusto estudo incluiu 12.813 pacientes hipertensos para comparar a eficácia terapêutica do BRA usado em monoterapia ou em combinação com outros anti-hipertensivos. Além disso, eles correlacionaram o efeito da PA com a meia-vida de cada BRA. O efeito sobre a PA foi avaliado pela avaliação da PA no consultório e pelo monitoramento domiciliar da PA (MRPA). Este último permite informações mais precisas sobre o efeito na PA a longo prazo de qualquer medicamento anti-hipertensivo. Em média, cada paciente obteve mais de 20 registros de PA ao longo de três dias de tratamento. Vale ressaltar que a prescrição era aberta a qualquer BRA a critério do médico. Como esperado, a losartana foi o BRA mais prescrito, tanto em monoterapia quanto em diferentes combinações. Apesar de ser o fármaco de menor custo entre os BRA, uma desvantagem é sua meiavida curta, exigindo intervalos menores entre os usos das pílulas, reduzindo assim a adesão ao tratamento. O estudo mostrou que as taxas de controle da PA foram maiores, tanto no consultório quanto na medida domiciliar, quando foi utilizado o BRA de vida mais longa. Como dito anteriormente, a taxa de anti-hipertensivos por pacientes no Brasil é razoável. O mesmo não pode ser dito em relação ao controle da PA, que ainda apresenta índices insuficientes,^6-8^ principalmente naqueles atendidos pelo sistema público de saúde e em uso de monoterapia, apesar das recomendações atuais,^4,7^ pois o mecanismo da hipertensão permanece desconhecido para a maioria dos pacientes.^4^

Os resultados apresentados por Barroso et al.,^9^ são importantes porque permitem duas conclusões principais. Uma delas tem impacto direto na abordagem terapêutica da hipertensão. Independentemente do BRA escolhido, este é mais eficaz para o controle da PA quando combinado com outras classes de anti-hipertensivos. A outra impacta nas políticas públicas de enfrentamento das DCNT apontando para a necessidade de avaliar a inclusão de pelo menos um BRA de meia-vida maior no SUS, melhorando o manejo da PA de pacientes hipertensos. Mesmo com medicamentos mais caros, níveis pressóricos mais baixos e estáveis são custo-efetivos, pois aumentam a prevenção de eventos que impactam negativamente na qualidade de vida e nos custos econômicos e sociais das DCV.
