# Supplementary material for: Diferenças entre os Bloqueadores dos Receptores da Angiotensina (BRA) no Tratamento da Hipertensão Arterial
Source: Arq Bras Cardiol. 2022 Jun 6;118(6):1083–4. [Article in Portuguese] doi: 10.36660/abc.20220281 (PMC9345143; doi:10.36660/abc.20220281)
Supplement: Translation

# Translation: Differences among Angiotensin Receptor Blockers (BRA) in the Treatment of Arterial Hypertension

**Authors:** José Geraldo Mill

**Affiliations:** 1 Vitória ES Brazil Departamento de Ciências Fisiológicas, Universidade Federal do Espírito Santo, Vitória, ES – Brazil; 2 Vitória ES Brazil Hospital Universitário Cassiano Antônio Moraes, Vitória, ES – Brazil

**Keywords:** Hypertension, Angiotensin-Converting Enzyme Inhibitors, Losartana, Early Diagnosis

Cardiovascular diseases (CVD) are the main cause of death and disability in Brazil, and arterial hypertension (AH) is the main risk factor for cardiovascular morbidity and mortality.^1^ Early diagnosis and correct treatment are priority actions to face the problem.^2^ The National Health Survey conducted by the Ministry of Health in 2013 (PNS-2013) determined the prevalence of AH by direct measurement of blood pressure (BP) and use of antihypertensive drugs in a representative sample of the Brazilian adult population. Prevalence of 32.3% (95%CI: 31.7 - 33.0) indicated nearly 50 million hypertensive patients.^3^ Around 70% depend on the Unified Health System (SUS) for both diagnosis and pharmaceutical care, an essential aspect of the Chronic Noncommunicable Diseases (NCD) plan.^2^

According to the current guidelines, the initial treatment of AH should be carried out with general measures, including regular aerobic physical activity, reduction of salt intake, increased consumption of fruits and vegetables and weight reduction when obesity or overweight is present.^4^ These measures benefit everyone and not only hypertensive patients indeed. Even adopting these strategies, many patients still depend on the regular use of drugs to get high BP control. Thus, the use of these drugs shows great importance because, given the dimension of the problem, even small pressure reductions generate a positive impact for millions of individuals affecting the morbidity and mortality rates due to CVD.^5^ Thus, the search for effective treatments for BP control has paramount importance to adopt public policies in this area.

The public health system provides at least one drug among the seven classes of antihypertensive medicines most often used in clinical routine, contributing to the high drug coverage in hypertensive patients in Brazil compared to other countries. A nationwide survey carried out in 2016 showed that 93.8% of individuals who knew their hypertensive state used at least one antihypertensive drug.^6^ High treatment indexes(>80%) were also reported in the PNS-2013 and in the ELSA-Brasil cohort, where most participants are attended by private health insurance.^7,8^ An important finding in the PNS was to show that the frequency of use was independent of schooling and income, confirming the universality of access, one of the objectives of the national policy to face CNCD in Brazil.^2^

Angiotensin receptor blockers (BRA) are Brazil’s most used antihypertensive drug.^7,8^ After the introduction of losartan, the prototype compound of BRA in the therapeutic arsenal of AH over 30 years ago, a series of other compounds with the same mechanism of action were available to use. The effectiveness of these compounds in BP control is the central theme of the article by Barroso et al.^9^ published in this issue of *Arquivos Brasileiros de Cardiologia*. This robust study included 12,813 hypertensive patients to compare the therapeutic efficacy of BRA used as monotherapy or in combination with other antihypertensive drugs. Additionally, they correlated the BP effect with the half-life of each BRA. The effect on BP was assessed by office BP assessment and by home BP monitoring (HBPM). The latter allows more accurate information on the long-term BP effect of any antihypertensive drug. On average, each patient obtained more than 20 BP records along three treatment days. It is worth mentioning that the prescription was open to any BRA at the doctor’s discretion. As expected, losartan was the most BRA prescribed, both as monotherapy and in different combinations. Despite being the drug with the lowest cost among BRA, one disadvantage is its short half-life, requiring shorter intervals between pill uses, thus reducing adherence to treatment. The study showed that the control rates of BP were higher, both in the office and in-home measurement, when longer-live ARB was used. As stated before, the rate of antihypertensive drugs by patients in Brazil is reasonable. The same cannot be said concerning BP control which still shows insufficient rates,^6-8^ mainly in those attended by the public health system and in use of monotherapy even though current recommendations^4,7^ since the mechanism of hypertension remains unknown for most patients.^4^

The results showed by Barroso et al.^9^ are important because they allow two main conclusions. One has a direct impact on the therapeutic approach to hypertension. Regardless of the BRA chosen, it is more effective for BP control when combined with other antihypertensive classes. The other impacts on the public policies for coping with CNCD point to the need to evaluate the inclusion of at least one longer half-life BRA in the SUS, improving the BP management of hypertensive patients. Even with more expensive drugs, lower and stable BP levels are cost-effective as they increase the prevention of events that negatively impact the quality of life and the economic and social costs of CVD
